# The effects of augmenting traditional rehabilitation with audio biofeedback in people with persistent imbalance following mild traumatic brain injury

**DOI:** 10.3389/fneur.2022.926691

**Published:** 2022-10-04

**Authors:** Kody R. Campbell, Robert J. Peterka, Peter C. Fino, Lucy Parrington, Jennifer L. Wilhelm, Natalie C. Pettigrew, Laurie A. King

**Affiliations:** ^1^Department of Neurology, Oregon Health and Science University, Portland, OR, United States; ^2^National Center for Rehabilitative Auditory Research, VA Portland Health Care System, Portland, OR, United States; ^3^Department of Health and Kinesiology, University of Utah, Salt Lake City, UT, United States; ^4^Department of Dietetics, Human Nutrition and Sport, La Trobe University, Melbourne, VIC, Australia

**Keywords:** mTBI, concussion, rehabilitation, biofeedback, sensory integration, sensorimotor, balance, vestibular

## Abstract

Complaints of non-resolving imbalance are common in individuals with chronic mild traumatic brain injury (mTBI). Vestibular rehabilitation therapy may be beneficial for this population. Additionally, wearable sensors can enable biofeedback, specifically audio biofeedback (ABF), and aid in retraining balance control mechanisms in people with balance impairments. In this study, we described the effectiveness of vestibular rehabilitation therapy with and without ABF to improve balance in people with chronic mTBI. Participants (*n* = 31; females = 22; mean age = 40.9 ± 11 y) with chronic (>3 months) mTBI symptoms of self-reported imbalance were randomized into vestibular rehabilitation with ABF (*n* = 16) or without ABF (*n* = 15). The intervention was a standard vestibular rehabilitation, with or without ABF, for 45 min biweekly for 6 weeks. The ABF intervention involved a smartphone that provided auditory feedback when postural sway was outside of predetermined equilibrium parameters. Participant's completed the Post-Concussion Symptom Scale (PCSS). Balance was assessed with the sensory organization test (SOT) and the Central Sensorimotor Integration test which measured sensory weighting, motor activation, and time delay with sway evoked by surface and/or visual surround tilts. Effect sizes (Hedge's G) were calculated on the change between pre-and post-rehabilitation scores. Both groups demonstrated similar medium effect-sized decreases in PCSS and large increases in SOT composite scores after rehabilitation. Effect sizes were minimal for increasing sensory weighting for both groups. The with ABF group showed a trend of larger effect sizes in increasing motor activation (with ABF = 0.75, without ABF = 0.22) and in decreasing time delay (with ABF = −0.77, without ABF = −0.52) relative to the without ABF group. Current clinical practice focuses primarily on sensory weighting. However, the evaluation and utilization of motor activation factors in vestibular rehabilitation, potentially with ABF, may provide a more complete assessment of recovery and improve outcomes.

## Introduction

Symptoms following a mild traumatic brain injury (mTBI) that persist for months after the initial injury can directly impact quality of life (1–3). Complaints of persistent imbalance are commonly reported after mTBI, with ~28% of people still reporting problems more than a year following their injury (4). Visual- and vestibular-related symptoms that occur after mTBI and can be predictive of long-lasting persistent symptoms well after the initial injury (5–7). However, recent evidence suggests that those with balance deficits due to a chronic mTBI (persistent mTBI symptoms >3 months after initial injury) have largely normal peripheral vestibular and ocular motor function (8). Therefore, symptoms of imbalance in people with chronic mTBI may be due to abnormal central processes, such as impaired sensory utilization, impaired sensory reweighting as conditions change, or sensorimotor control properties (9, 10).

Vestibular rehabilitation is an emerging treatment option for persistent imbalance and dizziness symptoms after mTBI (11–16). Recent evidence has demonstrated that rehabilitation programs that incorporate aspects of vestibular rehabilitation can decrease mTBI related symptoms and promote a faster return to activity (15, 17). However, recent systematic reviews highlighted that evidence for vestibular rehabilitation is currently lacking with few published randomized controlled trials (11, 18). The general exercise framework for vestibular rehabilitation after mTBI was adapted from other populations (19). A general vestibular rehabilitation program incorporates habituation exercises for impaired motion sensitivity, adaptation or gaze stability exercises for vestibulo-ocular reflex dysfunction, substitution exercises to promote central coordination of pre-planned eye movements, and static and dynamic balance exercises (14). Despite the name, vestibular rehabilitation does not exclusively train vestibular function—habituation, adaptation, and substitution exercises emphasize central sensory training and adaptations to reduce dizziness symptoms and improve balance (20).

A novel and emerging approach to vestibular rehabilitation is using biofeedback (21–23). Biofeedback provided during rehabilitation is a well-established technique that is grounded in theories of motor learning and may provide additional measures of performance that could tap into and change the motor control of balance (21, 24). Evidence from a recent systematic review of randomized controlled trials suggested that wearable sensors that provide biofeedback during rehabilitation can be effective in improving both static and dynamic forms of balance in populations with various neurological injury or disease (22). Visual and auditory biofeedback signals were the most commonly reported biofeedback modes identified by the systematic review. Audio biofeedback (ABF) can be utilized during balance exercises to provide concurrent measures of postural sway performance through changes in auditory pitch or location of tone, and ABF is well-suited for the mTBI population because the auditory cue does not interfere with other postural sensory systems, such as vision (25, 26). ABF also provides an additional sensory cue to coordinate balance (21, 27). ABF use has shown promise in improving postural sway while maintaining static balance in several patient populations including people with bilateral vestibular loss (25, 26) and cerebellar ataxia (28). Currently, there is a lack of research regarding the use of any biofeedback signal to augment rehabilitation for people with mTBI.

Given the potential benefit to enhance rehabilitation of imbalance in the chronic mTBI population through the addition of biofeedback during training, this study sought to explore the effects of augmenting vestibular rehabilitation with ABF relative to a standard vestibular rehabilitation program without ABF. We hypothesized that the addition of ABF would enhance the effectiveness of a standard vestibular rehabilitation program by reducing symptoms and improving balance control.

## Materials and methods

### Participants

All study participants provided written informed consent and the Oregon Health & Science University and Veterans Affairs Portland Health Care System joint Institutional Review Board approved recruitment procedures and experimental protocols. People who sustained an mTBI >3 months prior and reported continued imbalance (*n* = 40) participated in the study. Demographic details are given in Table 1. These participants were recruited for a larger study aimed at assessing central sensorimotor impairments after mTBI (NCT02748109). Participants were included if they: (1) were between 18 and 60 years old, (2) had minimal-to-no cognitive deficits as indicated by a Short Blessed Test score ≤8, and (3) were >3 months following a physician diagnosed mTBI with unresolved balance complaints. Participants were excluded if they had: (1) a previous or current musculoskeletal injury, surgery, medication, or neurological illness that would influence balance, (2) a pre-existing peripheral vestibular/oculomotor pathology, (3) have significant hearing loss that would interfere with the use of the ABF device; hearing loss no worse than 30 decibel (dB) HL (PTA 0.5–3 kHz), in the better ear, with the difference in ears being <15 dB PTA, or (4) moderate to severe substance abuse. Additional details for broader study inclusion/exclusion criteria, protocol, definitions for mTBI, and mTBI diagnosis confirmation can be found in Fino et al. (23).

**Table 1:** Demographics for participants receiving vestibular rehabilitation with and without auditory biofeedback (ABF).

**Variable**	**Without ABF**	**With ABF**	**Test Statistic**	***P*** **Value**
Enrolled (N)	20	20		
Completed rehabilitation (N)	15	16		
Withdrawing from study (N)	5	4		
Age (years)	40.2 (11.2)	39.5 (11.5)	0.642	0.526
Sex (male/female)	4/11	5/11	0.790^b^	0.779
Height (cm)	170.2 (10.3)	172.6 (8.5)	−0.707	0.485
Mass (kg)	75.8 (21.4)	82.9 (19.3)	−0.997	0.338
Days since injury^a^	366 (202, 658)	488 (232, 886)	225.0^c^	0.553
Pre-rehabilitation PCSS total symptom severity score	***44.9 (23)***	***27.9 (17.6)***	***2.332***	***0.027***
Pre-rehabilitation DHI total score	47.5 (21.6)	40.5 (19.8)	0.938	0.356
Rehabilitation compliance (%)^a^	92 (83, 100)	96 (73, 100)	252.5^c^	0.822
Home exercise program compliance (%)^a^	80 (43, 99)	88 (74, 96)	206.5^c^	0.661

Values are presented as mean and standard deviations unless noted otherwise. Independent *T*-tests were used to compare group demographics unless noted otherwise. Italicized and bolded values indicate significant group difference (*p* < 0.05).

a Data presented as median with 1st and 3rd quartile;

b Chi-Squared test;

c Wilcoxon Rank Sum test; PCSS, Post-Concussion Symptom Scale; DHI, Dizziness Handicap Index.

### Intervention

Participants in our larger study (NCT02748109) who qualified and were interested in rehabilitation for their symptoms were randomized to either vestibular rehabilitation with ABF or vestibular rehabilitation only (without ABF). Randomization occurred after enrollment and pre-rehabilitation assessments. Vestibular rehabilitation consisted of biweekly sessions for 6 weeks and included a warm-up with 5 min of walking, followed by 20 min of static balance exercises, and 20 min of dynamic balance exercises (Table 2). Each exercise was performed for 30 s and an additional 5 s-calibration period for the ABF system prior to performing the exercise. Subjects completed exercises sequentially and were given rest breaks as needed based on reported symptoms. To document improved tolerance to the exercises, the percentage of exercises completed out of the total number of exercises possible was calculated during each rehabilitation session. All subjects were asked to complete a home exercise program on the days they did not come to rehabilitation. The home exercise program included 2 sets of 45 s of gaze stabilization for vestibulo-ocular reflex, saccades, and visual motion sensitivity for both horizontal and vertical movements. Subjects were also given one habituation exercise to perform based on self-reported problems for 2 sets of 45 s that included seated head movements in a diagonal plane, horizontal head movements with the head tilted at 30 degrees flexion, vertical head movements with the head rotated at 45 degrees, or sit to stand with head in various positions. In addition, subjects were instructed to perform 15–20 min of cardiovascular exercise without increasing overall mTBI symptom severity by more than 2 points on a 10-point scale. If subjects increased symptom severity during exercise beyond 2 points they were told to stop the cardiovascular exercise (see Supplementary material for prescribed home exercise sheet). Subjects were provided a chart to track exercise adherence that was reviewed at each rehabilitation session.

**Table 2:** Exercises performed during vestibular rehabilitation sessions for with and without ABF groups.

	**Eyes open**	**Eyes closed**
	**Feet together—Firm surface**	
	1) Standing still 2) Tossing ball 3) Rotating head (H/ V) 4) Smooth pursuit (H/ V) 5) Gaze stabilization (H/ V)	1) Standing still 2) Rotating head (H/ V)
**Static**	6) Saccades (H/ V)	
	**Feet together—Foam surface**	
	1) Standing still 2) Tossing ball 3) Rotating head (H/ V)	1) Standing still 2) Rotating head (H/ V)
	**Tandem gait—Firm surface**	
	1) Walking 2) Tossing ball 3) Rotating head (H/ V)	1) Walking
**Dynamic**	**Tandem gait—Foam surface**	
	1) Walking 2) Tossing ball 3) Rotating head (H/ V)	1) Walking
	1) Chair	1) Chair
**Bending**	2) Side of treadmill	2) Side of treadmill
	3) Floor	3) Floor
	**Firm surface**	
	1) Sit-to-stand (mini squat) 2) Lunge 3) Lunge + twist	1) Sit-to-stand (mini squat) 2) Lunge 3) Lunge + twist
**Squatting**	**Foam surface**	
	1) Sit-to-stand (mini squat) 2) Lunge 3) Lunge + twist	1) Sit-to-stand (mini squat) 2) Lunge 3) Lunge + twist

H, horizontal; V, vertical.

Participants randomized to the ABF intervention wore a pair of headphones and a smartphone (Samsung Galaxy S4, Samsung Electronics Co., Suwon-si, South Korea) that was placed on the subject's back at L4/5 for static balance exercises and was raised to T7/8 for dynamic balance exercises during exercises at the rehabilitation sessions (25). The smartphone ABF application (uABF v1.0, 2016, mHealth Inc.) detected the linear acceleration of the smartphone to estimate body sway and provided feedback about sway acceleration as an auditory tone heard through the headphones. The ABF system provided feedback about mediolateral (ML) sway by changing the location of the tone (e.g., sway to the right would be encoded as tone heard through the right headphone); the system provided feedback about anteroposterior (AP) sway by changing the pitch of the tone (e.g., forward sway would be encoded as a high-pitched tone). The system was calibrated during 5 s of standing before each balance exercise to determine the limits used in the biofeedback algorithm based on the standard deviation (SD) of sway during the calibration. These calibration limits were used to map the acceleration values to the corresponding audio pitch and location of the tone. During standing conditions, the ABF system continuously provided feedback with limits of 2 SDs in the anterior direction, 1.5 SDs in the posterior direction, and 2 SDs in both right and left directions. When the participant was within the calibrated limits, there was no sound/feedback provided indicating stability. Feedback was provided when the person was outside of the calibrated limits. For example, the pitch was modulated on a linear scale ranging from no modulation (when the instantaneous acceleration was equal to the mean of the calibration period) to the highest pitch (when the instantaneous anterior acceleration was greater than or equal to the mean plus 2 SDs of the acceleration recorded during the calibration period) or to the lowest pitch (when the instantaneous posterior acceleration was greater than or equal to the mean plus 1.5 SDs of the acceleration recorded during the calibration period). A similar linear scale was implemented to shift the feedback between the right and left headphone; the tone was delivered equally in both ears when the ML acceleration was equal to the mean during the calibration period and ranged from only being delivered in the right ear (when the instantaneous acceleration to the right was greater than or equal to the mean plus 2 SDs of the acceleration during the calibration period) to only being delivered in the left ear (when the instantaneous acceleration to the left was greater than or equal to the mean plus 2 SDs of the acceleration during the calibration period), with a linear scale between the two. For example, a participant would hear a high-pitched tone in only their right ear if they were leaning forward and to the right beyond the calibration limits. During the walking exercises, the system was raised to the T7/8 level to provide feedback on the motion of upper trunk control. During walking tasks, feedback was only provided in the ML direction and was linearly scaled up to a limit of 10 SDs of the calibration period in both right and left directions. Additionally, feedback was not delivered during the walking exercises if the recorded acceleration was within 3 SDs of the mean of the calibration period. As no feedback was delivered based on the AP acceleration, there was no modulation of the pitch for the walking trials; only the location of the feedback (left or right ear) was modulated. Participants randomized to the ABF intervention wore the ABF device at the biweekly sessions for both static and dynamic exercises and completed the same exercises as the group without ABF. Participants in the ABF group received instructions on how to use the ABF device at each rehabilitation session. The participants were explained the auditory feedback tones and locations, provided through headphones, when the participant's sway, measured by the smartphone, exceeded the baseline calibration limits. Participants were instructed how to correct their balance and return to a stable, centered position during quiet stance according to the pitch and location of auditory biofeedback cues. The participants were also instructed on how to make corrections to ML trunk sway during gait. The ABF group did not do any additional exercises to become familiarized with the device. Home exercises were the same across groups and were not augmented with ABF in either group.

### Protocol

All participants provided baseline demographic information (age, sex, height, mass, days since injury) and completed questionnaires on post-mTBI symptom severity [Post-Concussion Symptom Scale (PCSS), taken from the Sport Concussion Assessment Tool Version 3], and on overall impact of dizziness on daily life (Dizziness Handicap Inventory—DHI). The PCSS assessed the severity of 22 concussion symptoms on a scale ranging from 0 (none) to 6 (very severe). The PCSS total symptom severity scores range from 0 (best) to 132 (worst). The DHI is a 25 item questionnaire that assessed the overall impact of dizziness on daily life (29, 30). Participants rated each item according to the perceived handicap caused by their dizziness using 0 (no handicap), 2 (sometimes), or 4 (always). The DHI total score ranges from 0 (no perceived handicap) to 100 (maximum perceived handicap). The PCSS and DHI were also assessed post-rehabilitation.

As part of the broader test battery, participants were evaluated on the sensory organization test (SOT) and a custom Central Sensorimotor Integration (CSMI) test pre- and post-rehabilitation (31, 32). Both tests were completed on a NeuroCom SMART Balance Master (Natus, California, USA). Participants performed SOT testing barefoot in a standardized foot position according to their height and completed six SOT conditions (31). Each condition lasted 20 s and was performed 3 times in sequential order. The SOT provided an equilibrium score ranging from 0 to 100 for each trial, with 100 indicating perfect stability and 0 indicating that the participant fell with testing halted during that trial. The composite score was calculated as a weighted average of the 6 conditions and was used as our outcome variable for a conventional balance performance measure.

All outcome measures of the CSMI test are obtained by a custom analysis applied to data recorded on a NeuroCom SMART Balance Master platform which had been modified to provide a higher contrast visual scene and to deliver low-amplitude (2° peak-to-peak) pseudorandom stimuli consisting of 12 continuously repeated 20-s duration cycles of wide bandwidth support surface-tilt and/or visual surround-tilt stimuli (9). Similar to the SOT, participants performed CSMI testing barefoot in a standardized foot position, according to their height, with their hands clasped in front and gaze forward. Participants performed 4 test conditions: (1) surface-tilt with eyes closed—SS/EC, (2) surface tilt with eyes open viewing a fixed visual surround—SS/EO, (3) visual surround tilt with eyes open with stance on a level surface—VS/EO, and (4) combined surface-tilt and visual-tilt with eyes open—SS + VS/EO. All conditions were performed with trials presented in randomized order and with rest breaks as needed. The AP center-of-mass (CoM) displacement was obtained by applying a phaseless 2nd order lowpass filter (cutoff frequency 0.469 Hz) to AP center-of-pressure measures from the NeuroCom forceplates. The AP CoM body sway angle was calculated trigonometrically from CoM displacement and the subject's CoM height measure (9).

A detailed description of the analysis of CSMI tests is provided elsewhere (9, 32) but briefly, frequency domain analyses were used to calculate an experimental frequency response function (FRF) which is the ratio of the across-cycle average discrete Fourier transform of the CoM sway angle to the across-cycle average discrete Fourier transform of the stimulus waveform (33). The FRF is comprised of gain values, representing the magnitude of the CoM response relative to the stimulus, and phase values, representing the timing of the CoM response relative to the stimulus, at 12 frequencies ranging from 0.05 to 1.5 Hz. The experimental FRF is compared to a model-predicted FRF with the model parameters adjusted using the Matlab “fmincon” function (Matlab version R2019b and Matlab Optimization Toolbox; The MathWorks Inc., Natick Massachusetts) to minimize the error between the experimental and model-predicted FRF.

The primary outcome measures of the CSMI test are parameters of a balance control model that account for the experimental FRF derived from the analysis of CoM sway evoked by the pseudorandom stimuli. The parameters included sensory weights, time delay, and motor activation factors. The sensory weights represent the relative contributions of proprioceptive, visual, and vestibular sensory information used for balance control such that, in each of the 4 test conditions, the sum of weights equaled 1. The time delay quantified the overall feedback delay due to neural transmission, central processing for sensory integration and motor command generation, and muscle activation. Motor activation is represented by two components (stiffness and damping factors) that determined how much corrective ankle joint torque was generated proportional to the sensory-derived internal estimate of body sway angular position and angular velocity, respectively. Stiffness and damping factors were normalized by dividing by the product of body mass, constant of gravitation, and CoM height to account for the high correlation of stiffness and damping factors with body mechanics (e.g., larger-sized participants require larger corrective ankle torques to compensate for the larger balance disturbance caused by gravity).

In addition to the balance control model parameters, we also calculated sway outcome measures of balance that included (1) the root mean squared (RMS) value of stimulus-evoked CoM sway, which is the RMS value of the zero-meaned, cycle-averaged, stimulus-evoked CoM sway angle data from the final 11 cycles of the pseudorandom stimulus, (2) the RMS value of the “remnant CoM sway” which is the square root of the sway variance that is not accounted for by the cycle-averaged sway response to the stimulus (33, 34), and (3) an estimate of the RMS value of the “internal sensory noise” that accounts for the measured remnant CoM sway using the assumption that the weighted summation of sensory orientation cues contributing to balance includes variability due to an internal sensory noise source.

### Statistical analysis

The with and without ABF group baseline demographic characteristics were compared with parametric and non-parametric group difference analyses. The outcome measures included the PCSS, DHI, and SOT composite scores, the model-derived parameters of the CSMI test (vestibular weights, time delay, normalized stiffness, and normalized damping), and CSMI sway-derived measures of balance performance. We limited analysis of the model-derived and sway-derived parameters of CSMI to conditions that directly evaluated vestibular weighting—SS/EC and SS + VS/EO conditions.

To describe the effects of augmenting rehabilitation with ABF on assessments of mTBI-symptoms and balance function, we calculated the effect size of the change between pre-and post-rehabilitation outcomes (Hedge's G) using the Measures of Effect Size (MES) Toolbox in MATLAB 2021b (MathWorks, Natick, MA). Additionally, we determined the number of participants that did and did not change their SOT composite score beyond a previously established minimal detectable change (MDC) score of 8 (35). Effect sizes were interpreted as: <0.19 as having little to no effect, 0.20–0.49 as a small effect, 0.50–0.79 as a medium effect, and >0.80 as a large effect (36).

## Results

### Participant characteristics

A total of 40 participants were enrolled and randomized for vestibular rehabilitation (20 with ABF and 20 without ABF) with 31 participants completing the rehabilitation and post-rehabilitation assessments (16 with ABF and 15 without ABF). Nine participants did not complete post-rehabilitation testing due to: pregnancy during the rehabilitation (*n* = 1), an unrelated rehabilitation musculoskeletal injury (*n* = 1), time constraints (*n* = 3), subject withdrawal due to randomized group assignment (*n* = 1), personal reasons (*n* = 1), and loss to follow up without communication (*n* = 2). Table 3 describes relevant demographic information between participants that did not complete the intervention and those included in the final analysis (Table 3).

**Table 3:** Demographics for participants completing vestibular rehabilitation and for those who withdrew from the intervention.

**Variable**	**Completed rehabilitation**	**Withdrawing from intervention**
*N*	31	9
Age (years)	40.9 (11.3)	40.3 (13.3)
Sex (male/female)	9/22	1/8
Days since injury^a^	393 (209, 733)	263 (212, 848)
Pre-rehabilitation PCSS total symptom severity score	36.1 (21.8)	37.0 (21.1)
Pre-rehabilitation DHI total score	43.9 (20.6)	39.1 (21.6)
Pre-rehabilitation SOT	55.5 (17.3)	57.4 (14.0)

Values are presented as mean and standard deviations unless noted otherwise.

a Data presented as median with 1st and 3rd quartile. PCSS, Post-Concussion Symptom Scale; DHI, Dizziness Handicap Index; SOT, Sensory Organization Test.

There were no significant group differences (with ABF and without ABF) on demographics, days since injury, pre-rehabilitation DHI, rehabilitation adherence, and home exercise adherence (Table 1). However, the ABF group reported a significantly lower PCSS symptom severity score at pre-rehabilitation relative to the without ABF group (Table 1). The time between pre- and post-rehabilitation evaluations was not different between the with and without ABF groups (mean ± SD for with ABF: 53 ± 3.5 days; without ABF: 52 ± 6 days, *t*_(22)_ = 0.56, *p* = 0.491). At the start of the intervention, participants in the without ABF and with ABF groups completed 29 and 32% of the exercises in the rehabilitation program, respectively. By the end of the intervention, participants in the without ABF and with ABF groups completed 65% and 60% of the exercises. However, both groups progressed similarly as indicated by the change in the percentage of the exercises completed over the intervention (with ABF: 36 ± 18%; without ABF: 28 ± 15%, Mann-Whitney *U* = 78.5, *p* = 0.104).

### Effects of augmenting vestibular rehabilitation with ABF on clinical balance performance measures

**Figure 1:**
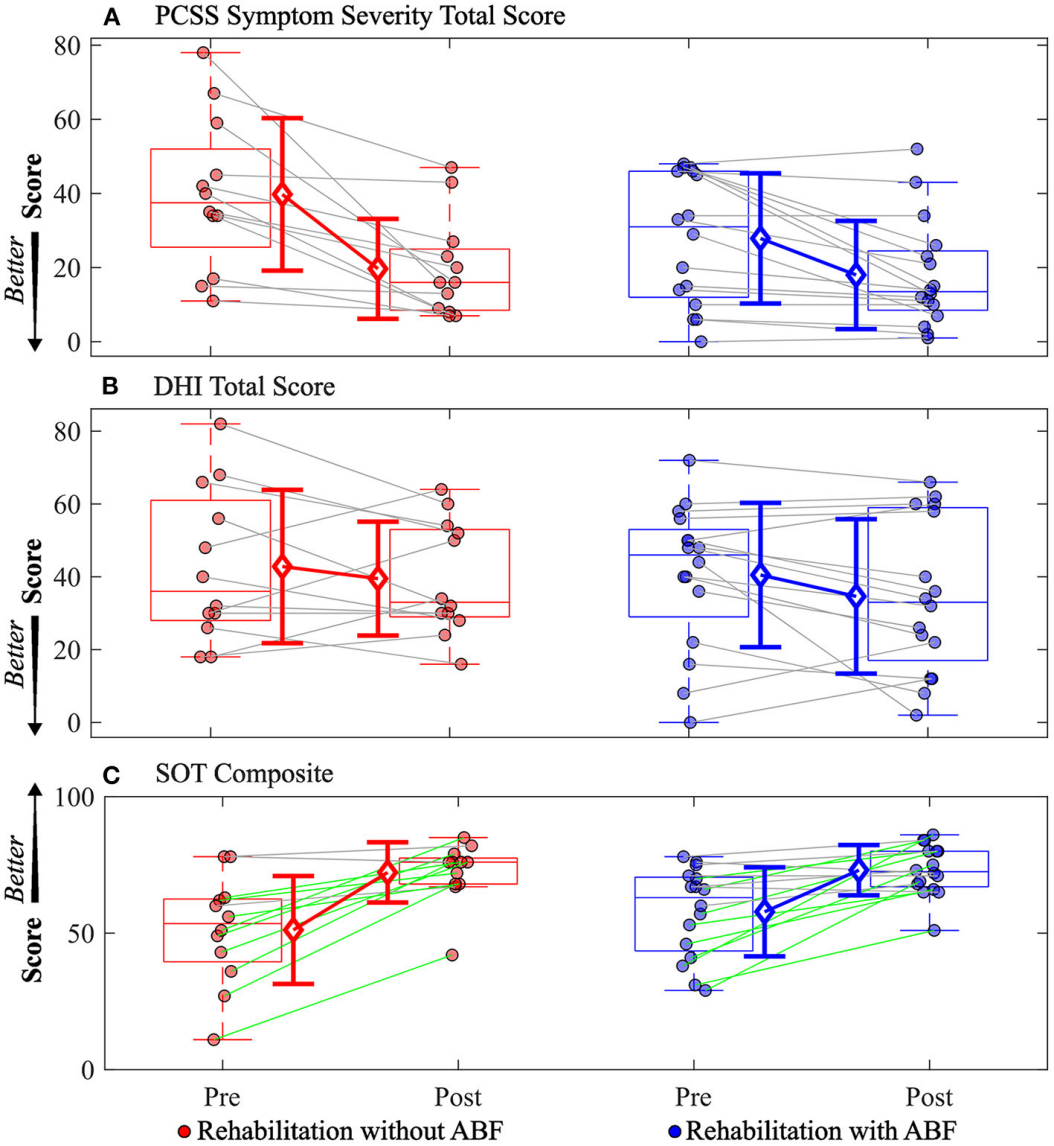
Individual participant data pre- and post-rehabilitation for groups without audio biofeedback (ABF—red) and with ABF (blue) for **(A)** Post-concussion Symptom Scale (PCSS) Symptom Severity total score, **(B)** Dizziness Handicap Index (DHI) total score, and **(C)** Sensory Organization Test (SOT) composite score. Central box shows median (center line) with upper and lower quartiles defined by 0.75 quantile and 0.25 quantile values, respectively. Whiskers extend to maximum and minimum values that are not considered outliers. Mean values are indicated by open diamonds with +/- 1 standard deviations. For the SOT composite score, green lines connecting participants indicate increased composite scores that were greater than the minimal detectable change of 8 as defined by a previous study (35); gray lines indicate composite scores were within the minimal detectable change range of +/- 8. Otherwise gray lines connect individual participant data.

Various trends emerged when comparing the rehabilitation groups with and without ABF. Both the with and without ABF groups had similar medium effect sizes in decreased PCSS total symptom severity scores (Table 4; Figure 1). However, the with ABF group demonstrated a small effect representing change on the DHI total score, while the without ABF group had no effect (Table 4; Figure 1). Both with and without ABF groups had large effects on increased SOT composite scores (Table 4; Figure 1). Additionally, both with and without ABF groups had a similar number of participants who improved their SOT composite score beyond the MDC value of 8 (without ABF = 10, with ABF = 9) or maintained (i.e., changes in SOT composite scores were within +/- 8) their SOT composite score (without ABF = 5, with ABF = 7). No participants from either group got worse on their SOT composite score beyond the MDC (<8).

**Table 4:** Means, standard deviations (SD), and effects sizes with lower and upper 95% confidence limits (Cl) for rehabilitation without and with audiobiofeedback (ABF) groups pre- and post-rehabilitation on self-reported (Post-Concussion Symptom Scale—PCSS; Dizziness Handicap Index—DHI), and objective (Sensory Organization Test -SOT; Central Sensorimotor Integration—CSMI) assessments of balance.

**Variable**	**Pre mean (SD)**		**Post mean (SD)**		**Effect size with lower and upper 95% CI**	
	**Without ABF**	**With ABF**	**Without ABF**	**With ABF**	**Without ABF**	**With ABF**
**Self-reported**						
PCCS total symptom severity score	44.9 (23)	27.9 (17.6)	27.5 (23.3)	18 (14.6)	−0.73^**^ (−1.52, −0.22)	−0.59^**^ (−1.16, −0.24)
DHI total score	47.5 (21.6)	40.5 (19.8)	45.3 (19)	34.6 (21.2)	−0.1 (−0.49, 0.26)	−0.28^*^ (−0.69, 0.01)
**Objective**						
SOT composite score	53.1 (18.6)	57.8 (16.3)	71.7 (12.1)	73.1 (9.2)	1.14^***^ (0.71, 2.02)	1.11^***^ (0.7, 1.72)
**CSMI—SS/EC**						
Vestibular weighting	0.432 (0.065)	0.494 (0.062)	0.468 (0.083)	0.498 (0.074)	0.46^*^ (−0.01, 1.14)	0.06 (−0.37, 0.56)
Time delay (ms)	166 (23)	168 (17)	155 (20)	155 (14)	−0.46^*^ (−1.16, −0.18)	−0.84^***^ (−1.45, −0.36)
Normalized stiffness	1.45 (0.18)	1.42 (0.12)	1.53 (0.19)	1.52 (0.1)	0.6^**^ (0.11, 1.24)	0.75^**^ (0.35, 1.35)
Normalized damping	0.487 (0.064)	0.502 (0.049)	0.523 (0.05)	0.542 (0.054)	0.41^*^ (0.18, 0.84)	0.83^***^ (0.31, 1.53)
**CSMI—SS + VS/EO**						
Vestibular weighting	0.412 (0.065)	0.448 (0.057)	0.428 (0.087)	0.451 (0.075)	0.19 (−0.25, 0.83)	0.05 (−0.62, 0.65)
Time delay (ms)	163 (26)	168 (25)	148 (26)	150 (20)	−0.52^**^ (−1.25, −0.17)	−0.77^**^ (−1.42, −0.28)
Normalized stiffness	1.41 (0.18)	1.37 (0.1)	1.45 (0.15)	1.46 (0.13)	0.22^*^ (−0.13, 0.8)	0.76^**^ (0.25, 1.41)
Normalized damping	0.466 (0.06)	0.453 (0.05)	0.483 (0.067)	0.498 (0.06)	0.25^*^ (−0.27, 0.97)	0.79^**^ (0.34, 1.4)

EC, eyes closed; EO, eyes open; SS, support surface stimulus; VS, visual surround stimulus;

*** Large effect;

** Medium effect;

* Small effect. Cell shading indicates the strength of effect size for a quick comparison with increasing shading darkness indicating larger effect sizes.

**Figure 2:**
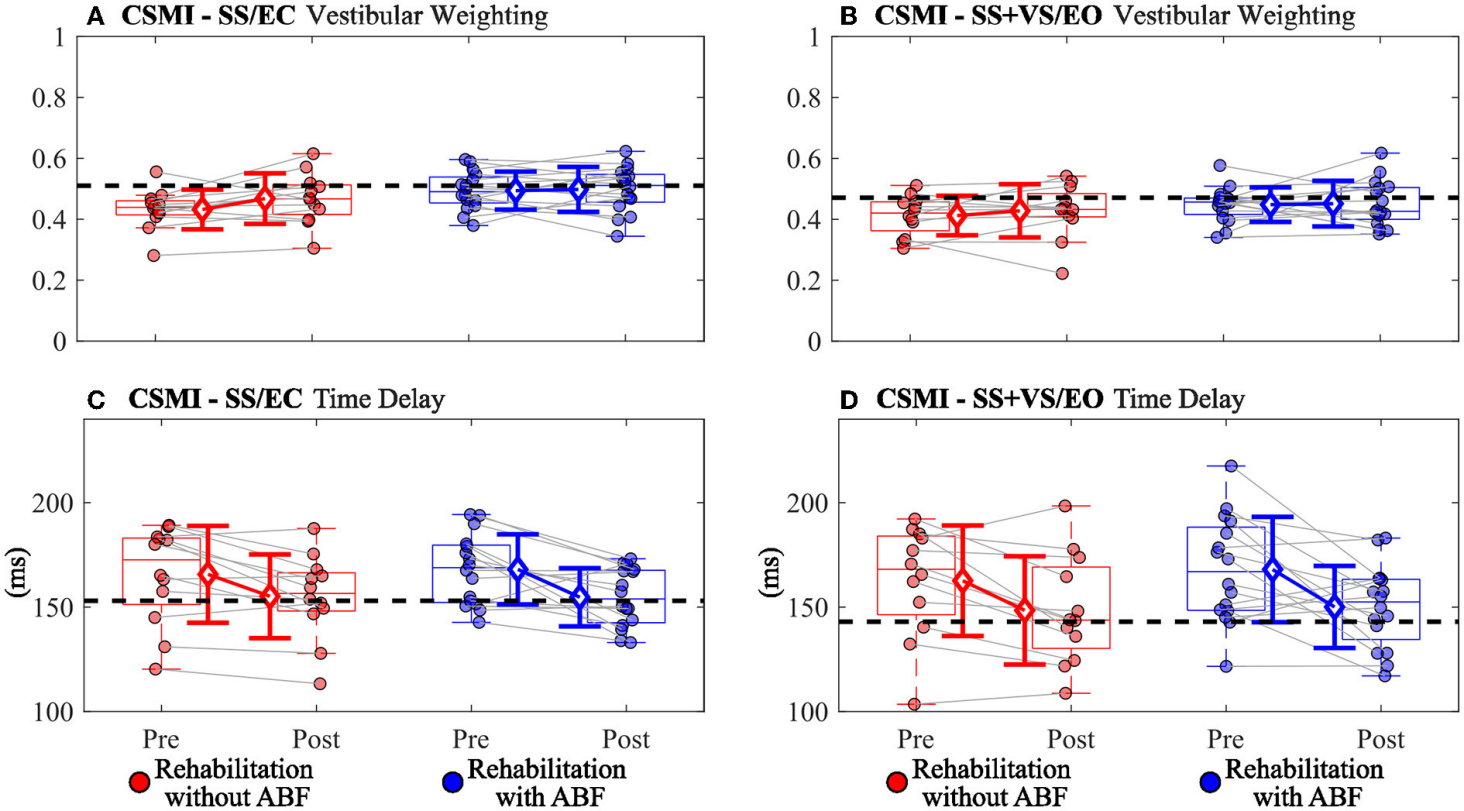
Individual participant data pre- and post-rehabilitation for groups without audio biofeedback (ABF—red) and with ABF (blue) for **(A)** vestibular weighting during surface-tilt with eyes closed (SS/EC), **(B)** vestibular weighting during combined surface-tilt and visual-tilt with eyes open (SS+VS/EO), **(C)** time delay during SS/EC, and **(D)** time delay during SS + VS/EO. Central box shows median (center line) with upper and lower quartiles defined by 0.75 quantile and 0.25 quantile values, respectively. Whiskers extend to maximum and minimum values that are not considered outliers. Mean group values are indicated by open diamonds with +/- 1 standard deviations. Gray lines connect individual participant data. Blacked dashed horizontal line indicates mean value derived from control subjects in Supplementary material from Peterka et al. (9). Values moving toward the healthy control mean value indicate improved performance.

**Figure 3:**
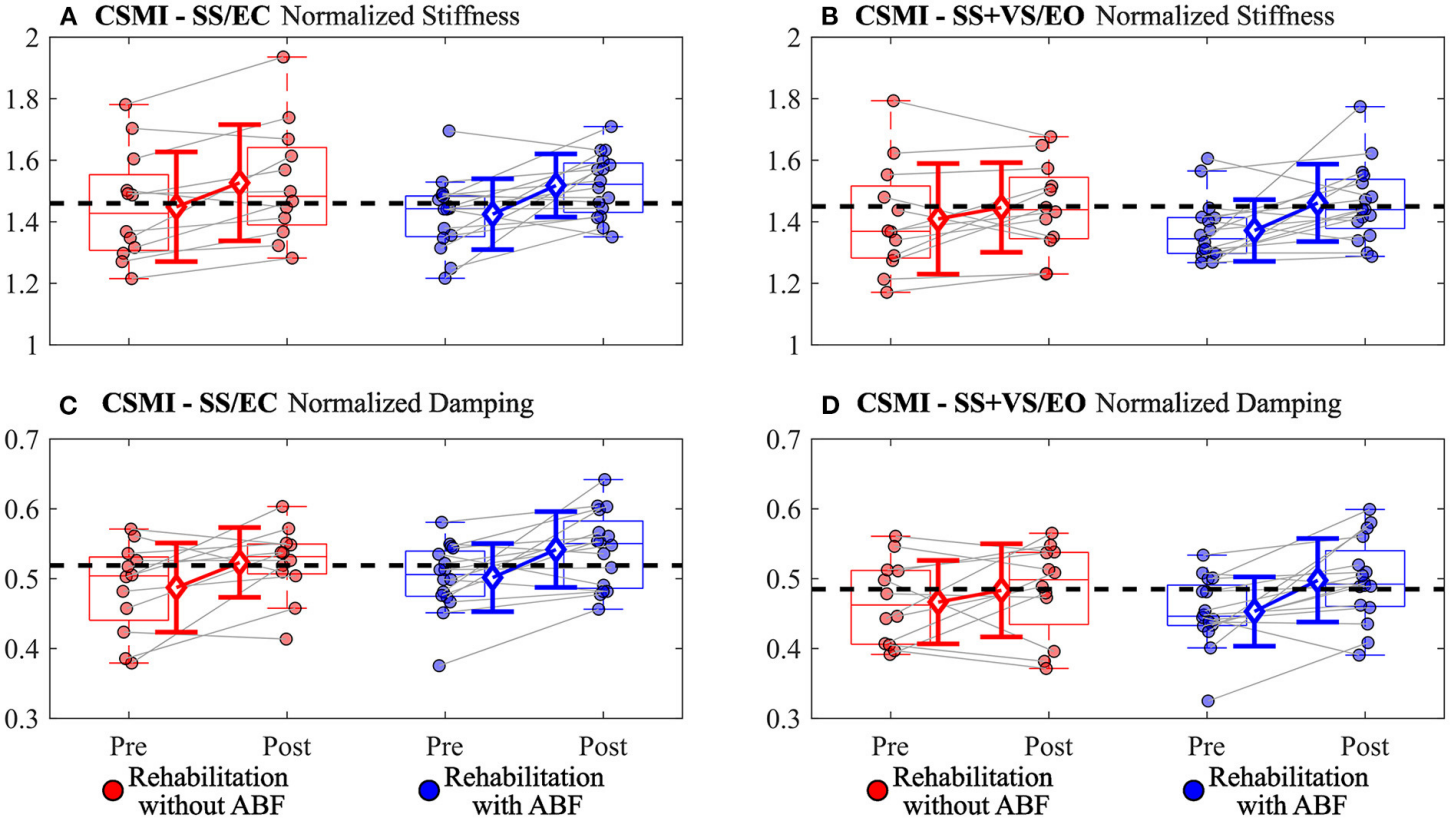
Individual participant data pre- and post-rehabilitation for groups without audio biofeedback (ABF—red) and with ABF (blue) for **(A)** normalized stiffness during surface-tilt with eyes closed (SS/EC), **(B)** normalized stiffness during combined surface-tilt and visual-tilt with eyes open (SS+VS/EO), **(C)** normalized damping during SS/EC, and **(D)** normalized damping SS + VS/EO. Central box shows median (center line) with upper and lower quartiles defined by 0.75 quantile and 0.25 quantile values, respectively. Whiskers extend to maximum and minimum values that are not considered outliers. Mean group values are indicated by open diamonds with +/- 1 standard deviations. Gray lines connect individual participant data. Blacked dashed line indicates mean value derived from control subjects in Supplementary material from Peterka et al. (9). Values moving toward the healthy control mean value indicate improved performance.

There were group-dependent trends in the magnitudes of effect sizes for the CSMI model-derived parameters and sway-derived outcomes. The group without ABF had small effects on changes in vestibular weighting, while the group with ABF had no effects (see effect sizes; Table 4; Figure 2). For the time delay parameter, the without ABF group had small to medium effects on time delay decreases, while the group with ABF had medium to large effects (see effect sizes; Table 4; Figure 2). Lastly, the group without ABF had small to medium increases in motor activation (normalized stiffness and damping) and the group with ABF had medium to large increases (see effect sizes; Table 4; Figure 3).

**Figure 4:**
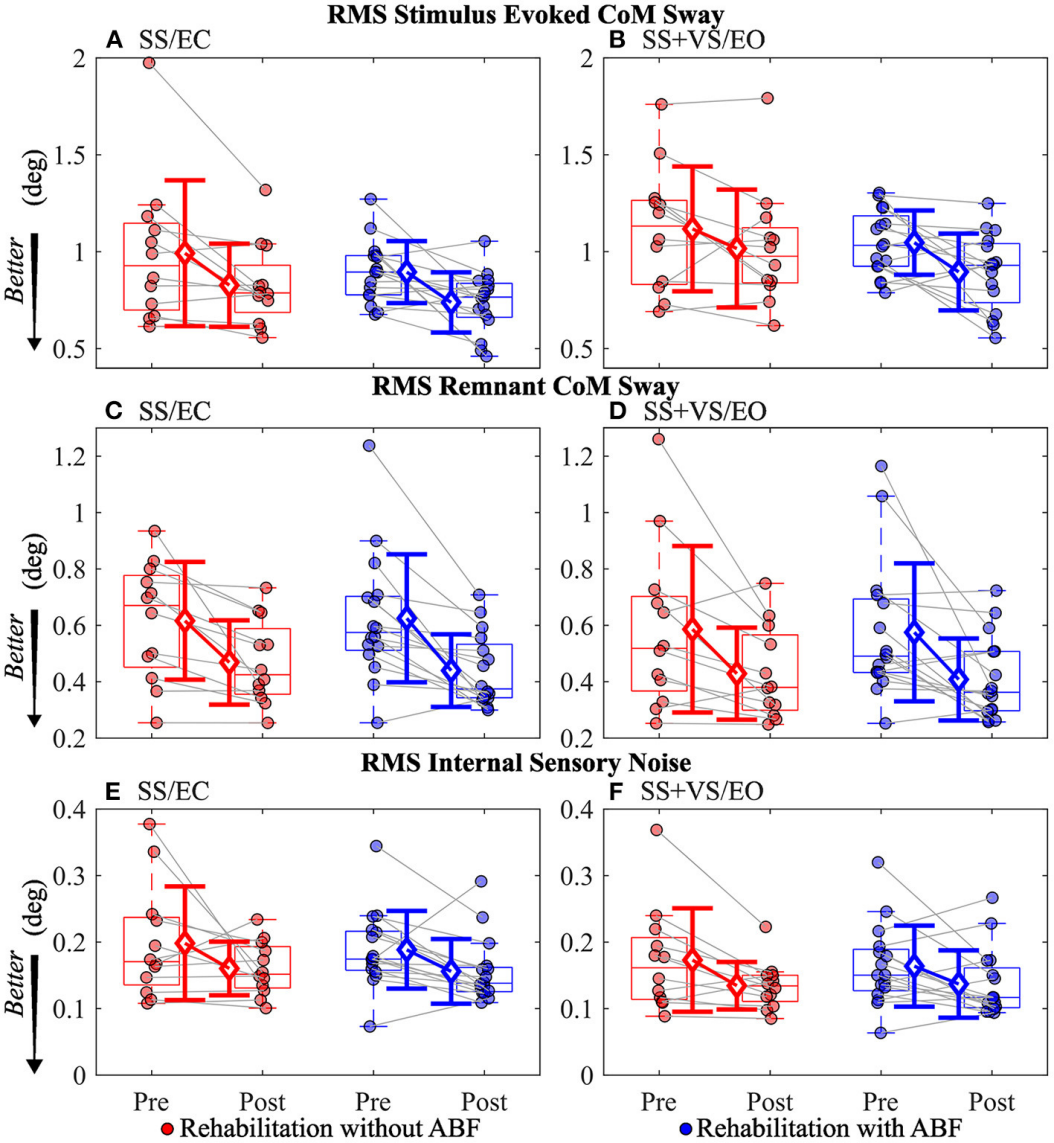
Individual participant data pre- and post-rehabilitation for groups without audio biofeedback (ABF—red) and with ABF (blue) for Root Mean Squared (RMS) stimulus evoked Center of Mass (CoM) sway during **(A)** surface-tilt with eyes closed (SS/EC) and **(B)** during combined surface-tilt and visual-tilt with eyes open (SS + VS/EO); RMS remnant CoM sway during **(C)** SS/EC and **(D)** SS+VS/EO; RMS internal sensory noise during **(E)** SS/EC and **(F)** SS + VS/EO. Central box shows median (center line) with upper and lower quartiles defined by 0.75 quantile and 0.25 quantile values, respectively. Whiskers extend to maximum and minimum values that are not considered outliers. Mean group values are indicated by open diamonds with +/- 1 standard deviations. Gray lines connect individual participant data.

For the stimulus-evoked RMS CoM sway, the group without ABF had small to moderate decreases, and the ABF group had large decreases (see effect sizes; Table 5; Figure 4). The group without ABF had medium effects on decreases in RMS remnant CoM sway, and the ABF group had large effects (see effect sizes; Table 5; Figure 4). Finally, we observed that the group without ABF had medium effects on decreases in RMS internal sensory noise and the group with ABF had small to medium effects (see effect sizes; Table 5; Figure 4).

**Table 5:** Means, standard deviations (SD), and effects sizes with lower and upper 95% confidence limits (Cl) for rehabilitation without and with audiobiofeedback (ABF) groups pre- and post-rehabilitation on Central Sensorimotor Integration (CSMI) test measures of sway.

**Variable**	**Pre mean (SD)**		**Post mean (SD)**		**Effect size with lower and upper 95% CI**	
	**Without ABF**	**With ABF**	**Without ABF**	**With ABF**	**Without ABF**	**With ABF**
**CSMI condition—SS/EC**						
RMS CoM sway	0.99 (0.38)	0.895 (0.16)	0.827 (0.214)	0.738 (0.155)	−0.51^**^ (−1.05, −0.24)	−0.96^***^ (−1.59, −0.46)
RMS remnant CoM sway	0.616 (0.209)	0.625 (0.227)	0.468 (0.149)	0.439 (0.129)	−0.78^**^ (−1.47, −0.39)	−0.97^***^ (−1.71, −0.61)
RMS internal sensory noise	0.198 (0.085)	0.188 (0.058)	0.16 (0.04)	0.156 (0.049)	−0.54^**^ (−1.15, 0.12)	−0.58^**^ (−1.48, −0.08)
**CSMI condition—SS + VS/EO**						
RMS CoM sway	1.12 (0.32)	1.05 (0.17)	1.02 (0.3)	0.895 (0.198)	−0.31^*^ (−0.98, 0.07)	−0.8^***^ (−1.47, −0.28)
RMS remnant CoM sway	0.586 (0.295)	0.575 (0.245)	0.429 (0.163)	0.408 (0.145)	−0.63^**^ (−1.1, −0.27)	−0.8^***^ (−1.29, −0.41)
RMS internal sensory noise	0.173 (0.078)	0.164 (0.061)	0.134 (0.036)	0.137 (0.051)	−0.61^**^ (−1.21, −0.29)	−0.46^*^ (−1.04, −0.02)

All sway and noise measures have units of degrees. EC, eyes closed; EO, eyes open; SS, support surface stimulus; VS, visual surround stimulus; RMS, root mean square; CoM, center of mass.

*** Large effect;

** Medium effect;

* Small effect. Cell shading indicates the strength of effect size for a quick comparison with increasing shading darkness indicating larger effect sizes.

## Discussion

Imbalance is common following mTBI and can persist for months after the initial injury. Furthermore, treatment can be difficult for this population because of the assumption that persistent mTBI symptoms and imbalance usually do not have a single etiology (37). In this study, we explored the effects of augmenting traditional vestibular rehabilitation with a novel ABF tool. On average and regardless of using ABF, participants decreased PCSS symptom severity scores and improved balance function after rehabilitation when measured with the SOT—a common objective assessment of balance. When balance was assessed with the CSMI test, which quantified the sensory weighting, time delay, and motor activation properties of the balance control system, different trends in the magnitudes of the effect sizes of change in the CSMI model-derived parameters and sway-derived measures emerged between the groups. Both groups improved on aspects of the balance control system including decreased time delay, increased motor activation, and decreased sway-derived measures. However, we observed a general trend of the ABF group having larger improvements in these measures. There were small-to-no changes in vestibular weighting for both rehabilitation groups. Nonetheless, this study adds to the growing literature supporting the use of vestibular rehabilitation for improving overall mTBI symptoms for acute, subacute, and chronic stages of recovery (15, 17, 38).

The vestibular rehabilitation used in this study was provided biweekly over 6 weeks. The frequency of therapy sessions may in part explain the positive results on overall mTBI symptom severity as this frequency is more often than what is typical in routine clinical care. A major goal of vestibular rehabilitation is to promote central nervous system compensation through exercise-derived techniques that incorporate adaptation, habituation, and substitution (20). This goal comes with the objective of reducing dizziness-related symptoms and/or imbalance (20). Contrary to the moderate-sized reductions we observed in overall mTBI symptoms following rehabilitation in both groups, we did not observe a similar magnitude of change after rehabilitation in the DHI. Two reasons may explain the difference in magnitudes of effect sizes of change between the PCSS and the DHI. The first reason is that the PCSS evaluates a broader range of mTBI-related symptoms that can include headache/migraine, cognitive, mood, vestibular/ocular motor, and sleep symptom clusters, while the DHI focuses on perceived handicaps due to dizziness alone (39–41). While the rehabilitation program was focused on aspects of vestibular rehabilitation, it is possible that unexpected changes or improvements occurred to mTBI-related symptom clusters (e.g., sleep or mood) that we would not expect to be associated with vestibular rehabilitation. Secondly, individual participant data showed a wide range of changes in DHI total scores from pre-rehabilitation to post-rehabilitation, with one participant reporting a 20 point increase (i.e., a worse/higher handicap) and another reporting a 42 point decrease (i.e., an improved/ lower handicap) after rehabilitation (Figure 1). Other studies have shown significant reductions in DHI total scores following some form of vestibular rehabilitation (15, 17). However, these studies were primarily performed on adolescents and young adults (<30 years old) during a more acute and subacute mTBI recovery phase, while the participants in our study had an average age of about 40 years and median time since injury was ~1 year.

While our study showed no considerable effect of vestibular rehabilitation for either group on DHI, it did demonstrate large improvements in postural stability as measured by the SOT in both groups. Significant SOT improvements were previously observed for people completing a home vestibular rehabilitation program (38). Improvements in the SOT, theoretically, represent changes in central sensory integration and processing. After rehabilitation for both groups in our study AP sway was decreased during SOT conditions relative to pre-rehabilitation assessments. However, the SOT paradigm evaluates postural stability based on condition-dependent sway, which may lead to incomplete conclusions about the changes in the balance control system that produced the reduced sway. The CSMI test was developed to provide a more complete assessment of the mechanisms contributing to balance control.

Although we found large but similar improvements in balance function using the SOT composite score as an outcome measure between groups, the CSMI test showed small to no changes in vestibular weighting for both groups (Table 4; Figure 2). The interpretation of what balance control mechanisms contribute to changes in outcome measures in SOT and CSMI testing differ greatly from one another. Altered or dysfunctional utilization of sensory information, assessed using condition-dependent measures of spontaneous sway, is assumed to be represented by the SOT score. In contrast, the CSMI test uses a stimulus-evoked sway assessment to evaluate both sensory utilization, represented by sensory weights, and central processing that leads to motor activation and corrective joint torque generation. Previous results have shown that the CSMI test provides valid measures of the vestibular contribution to balance. Specifically, CSMI results in subjects with bilateral vestibular loss show essentially zero values for vestibular weights (32), and reduced vestibular weights in subjects with complete unilateral vestibular loss (42).

Another difference between the SOT and CSMI is that SOT testing presents sudden changes in the availability of sensory cues that require a subject to quickly reweight their sensory utilization to prevent instability. In contrast, CSMI testing is performed in steady state conditions where subjects have time to adjust to different test conditions. Thus, SOT outcome scores are influenced by a subject's reweighting ability in addition to the availability of sensory information. Finally, the SOT does not assess how the motor aspects of balance control might influence the levels of spontaneous sway recorded in the SOT test conditions.

Increases in vestibular weighting were only observed in the rehabilitation group without ABF in the SS/EC condition and this increase was small while there was essentially no difference between pre- and post-rehabilitation vestibular weighting in the SS + VS/EO condition and in the with ABF group in the SS/EC condition (Table 4). A possible explanation for the vestibular weight increase in the without ABF group in the SS/EC is that, on average, the pre-rehabilitation vestibular weight was lower compared to the group that used ABF. Additionally, the average weight was lower for the without ABF group than the average vestibular weight in control subjects from a previous study using CSMI methods (9), while the ABF group had similar vestibular weights relative to the control subjects (Figure 2). Therefore if a vestibular weight is low, rehabilitation without ABF, and possibly with ABF, may have the ability to increase vestibular utilization. But if vestibular weighting already is appropriate for a given test condition the rehabilitation may not change vestibular weighting.

The ABF group had larger magnitudes of effect sizes in changes to time delay and motor activation measures compared to the without ABF group. The immediate feedback on postural sway performance during balance exercises provided by ABF appeared to have some benefit relative to the vestibular therapy without ABF in improving time delay and motor activation. The decreased time delay after rehabilitation suggests that participants were faster or more efficient in generating postural corrections to balance. Auditory cues can be processed at fast rates, and can be combined with other senses for postural control to help decrease postural sway during standing balance (27). The additional sensory cue for deriving body orientation and immediate performance feedback in the ABF group may have led to subjects becoming accustomed to making faster corrections and, thus, possibly explaining the decreased post-rehabilitation time delays we observed.

In a closed-loop feedback control system, like the balance control system, longer time delays are detrimental to system stability (32). An appropriate compensation for an increased time delay is to reduce motor activation stiffness and damping factors. However, the consequence of reduced motor activation is increased sensitivity to balance perturbations, consistent with the relatively large RMS stimulus-evoked CoM sway and remnant CoM sway values on the pre-rehabilitation CSMI tests. Increased sensitivity arises because the reduced motor activation allows the destabilizing torque due to gravity to have a greater influence on body sway. We speculate that the time delay reduction may be a key factor affected by rehabilitation since reduced time delay would allow the balance control system to increase motor activation without jeopardizing stability and thus restoring more normal balance control behavior.

The CSMI model-based interpretation of experimental stimulus-evoked sway provided an estimate of internal sensory noise which is the presumed major source of spontaneous sway variability measured during unperturbed stance and is the source of variability that produced the remnant CoM sway that we measured (34). The remnant CoM sway is a function of the internal sensory noise and is affected by motor activation such that lower motor activation results in larger remnant CoM sway for a given internal sensory noise magnitude. Both groups showed reductions in the internal sensory noise and the effect sizes were similar in both groups (Table 5; Figure 4). A reduction in internal sensory noise may indicate that vestibular rehabilitation resulted in a more consistent or reliable central processing of sensory information needed for balance control.

Both without ABF and with ABF vestibular rehabilitation groups showed reductions in time delay, increases in motor activations, and consistent with these changes, reductions in stimulus-evoked and remnant CoM sway. However, the effect size changes were consistently larger in the with ABF group compared to the without ABF group (Table 4; Figures 2–4). This is an important finding because time delay and motor activation are not measured during routine clinical care. Vestibular rehabilitation could potentially be modified to include exercises that might be more effective in modifying time delay and motor activation rather than focusing solely on exercises designed to improve sensory integration and reweighting. Nonetheless, at this stage, further work and evidence are required to confirm our results and our interpretations.

While it was not a main objective of the current paper, there remains a question if changes in subjectively reported mTBI symptom severity or dizziness-perceived handicap from rehabilitation relate to changes in objectively measured balance performance, measured through the SOT or the CSMI test. When considering both the with and without ABF groups together, *post-hoc* correlation analyses showed no significant relationships between changes in PCSS evaluations with changes in objective balance measurements and only one significant relationship for changes in DHI (Supplementary Table 1). Additionally, there were no significant relationships between pre-rehabilitation measures of PCSS total symptoms with SOT and CSMI measures for all participants regardless of group and only a limited number of significant correlations of DHI with CSMI measures in the SS/EC condition (Supplementary Table 2). Our results add to the conflicting research on the relationships between self-reported mTBI symptoms with balance performance using clinically rated or instrumented measurements (8, 43–45). However, a recent study showed that mTBI severity, quantified by a single factor from the shared variance of multiple questionnaires (DHI, PCSS, and Neurobehavioral Symptom Inventory) was directly related to balance impairment, which was quantified by a balance sway dispersion factor comprised from the shared variance of RMS sway from multiple stance (double stance vs. single leg) and visual conditions (eyes open or closed) measured with wearable sensors (46). This study also showed, through mediation analyses, that the level of balance impairment was mediated by factors of motor activation and time delay, similar to the measures quantified in this study (46). A similar mediation analysis approach could reveal relationships between mTBI symptom/injury severity change with change in balance performance and could be the starting point of better understanding if there are underlying physiological contributions to changes in self-perceived function.

Our study is not without limitations. We had a relatively low sample size for the study and had a high dropout rate (~25%). However, this study was an exploratory aim of a larger study aimed at assessing central sensory impairments in people with chronic mTBI symptoms (23). Our data and observations provide important information to support additional work in novel treatment methods for improving balance performance and function following mTBI. Additionally, our results may support a shift in the treatment goal for the chronic mTBI population group that combines central sensory integration with neuromuscular training. Attrition bias, which could occur if participants with more severe balance impairments dropped out of the study, was initially a concern. However, those that did and did not complete the intervention were similar in aspects of age, sex, days since injury, and pre-rehabilitation scores of PCSS, DHI, and SOT (Table 3). In the context of the study sample, there was a higher proportion of females (59%) compared to males (41%) that completed rehabilitation with and without ABF. However, there was no difference in the proportion of females and males between the ABF groups (Table 1). Biological sex and gender are important psychosocial biological variables that can influence mTBI injury risk, symptom presentation, recovery patterns, and can even influence points of entry into the medical system as well as referral patterns (47–49). Given the role of biological sex and gender, our results should be interpreted in the context of our study sample and may not extend to other studies with different proportions of males and females.

Another limitation is that our results focused on static balance performance. Therefore, the effects of vestibular rehabilitation with and without ABF on dynamic balance (e.g., gait) remain unknown. There are differences in the physiological mechanisms required for standing balance and dynamic balance. Therefore, future work should explore the effectiveness of rehabilitation with and without ABF on dynamic balance since dynamic balance is particularly relevant for daily life function and navigating in the real world. Lastly, although we observed consistent and large improvements in symptom severity and some aspects of sensory integration, our research protocol of biweekly sessions does not reflect current clinical practice. Patients are typically only seen by a therapist once or twice and are expected to perform exercises at home without supervision. Future studies should investigate dose-response parameters to determine optimal scheduling of therapy.

## Conclusion

Vestibular rehabilitation augmented with and without ABF was able to decrease persistent post-mTBI symptom severity in people on average a year from their initial mTBI. Additionally, both rehabilitation with and without ABF improved balance function when measured with the SOT. However, larger improvements in time delay and motor activation components of balance function were observed for people completing vestibular rehabilitation augmented with ABF. These preliminary results suggest additional approaches such as neuromuscular training and real-time biofeedback should be considered for rehabilitation after mTBI.

## Data availability statement

The raw data supporting the conclusions of this article will be made available by the authors, without undue reservation.

## Ethics statement

The studies involving human participants were reviewed and approved by Oregon Health and Science University and Veterans Affairs Portland Health Care System joint Institutional Review Board. The patients/participants provided their written informed consent to participate in this study.

## Author contributions

LK and RP contributed to the conception and design of this study. KC, LP, PF, and RP contributed to data acquisition. JW and NP provided subject rehabilitation. KC, LK, LP, PF, and RP contributed to analysis and interpretation of data. KC, RP, JW, NC, and PF wrote the first draft of the manuscript. All authors contributed to the manuscript revision, read, and approved the submitted version.

## Funding

This work was supported by the Assistant Secretary of Defense for Health Affairs (Award Nos. W81XWH-15-1-0620 and W81XWH-17-1-0424).

## Conflict of interest

The authors declare that the research was conducted in the absence of any commercial or financial relationships that could be construed as a potential conflict of interest.

## Publisher's note

All claims expressed in this article are solely those of the authors and do not necessarily represent those of their affiliated organizations, or those of the publisher, the editors and the reviewers. Any product that may be evaluated in this article, or claim that may be made by its manufacturer, is not guaranteed or endorsed by the publisher.

## Author disclaimer

Opinions, interpretations, conclusions, and recommendations are those of the author and are not necessarily endorsed by the Department of Defense.
