# Supplementary material for: The effects of augmenting traditional rehabilitation with audio biofeedback in people with persistent imbalance following mild traumatic brain injury
Source: Front Neurol. 2022 Oct 4;13:926691. doi: 10.3389/fneur.2022.926691 (PMC9577092; doi:10.3389/fneur.2022.926691)
Supplement: Supplementary file 1 [file Data_Sheet_1.docx]

Supplementary Material

# Home exercise program provided to participants

Home Exercise Program

Perform each of the 5 exercises once a day, on the days you do not come in for therapy.

**For Exercises 1-3:**

- Perform while seated
- Target = tip of pen held at arm’s distance


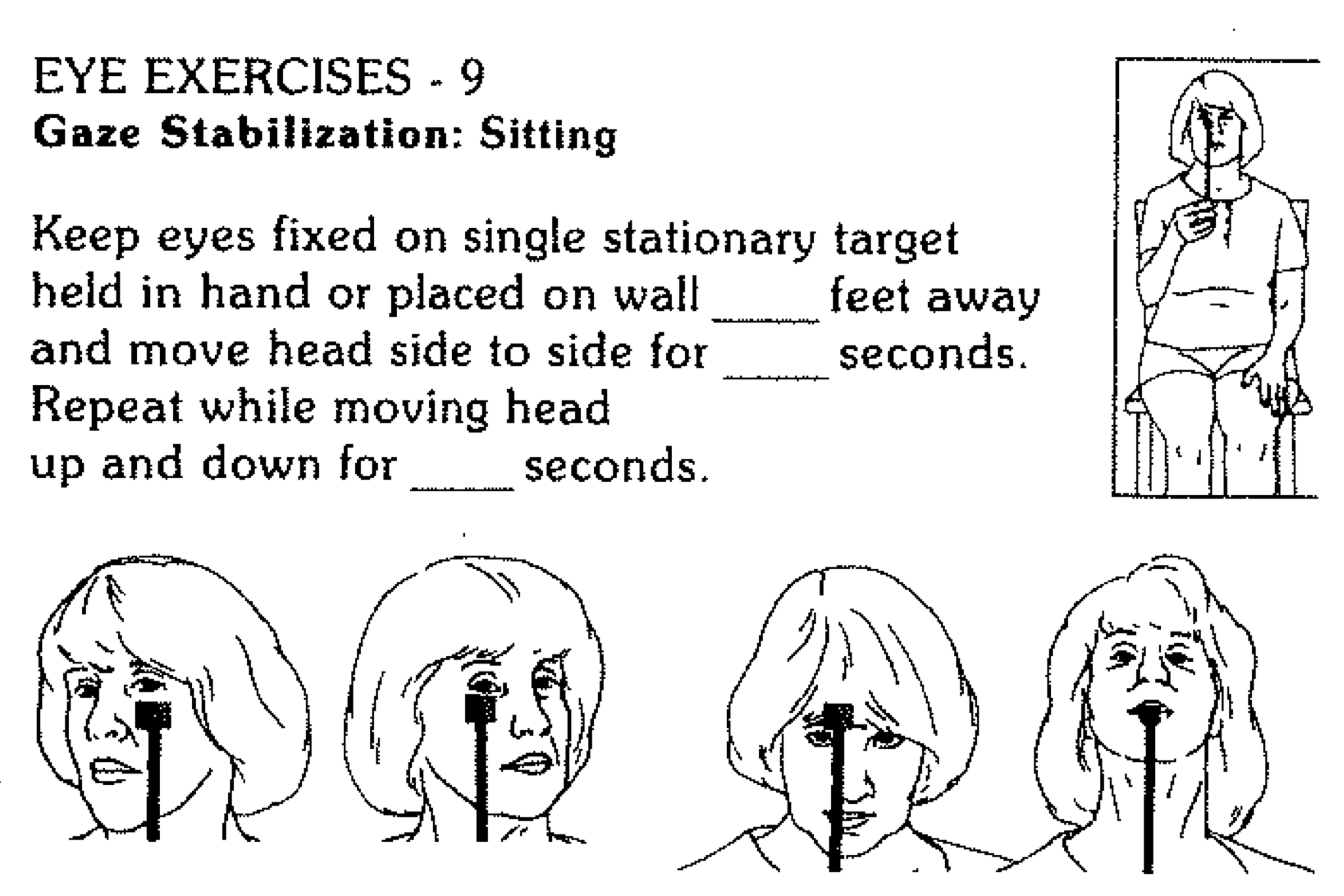


**Exercise #1 (Gaze Stabilization):**

- 2 x 45 sec side/side
- 2 x 45 sec up/down


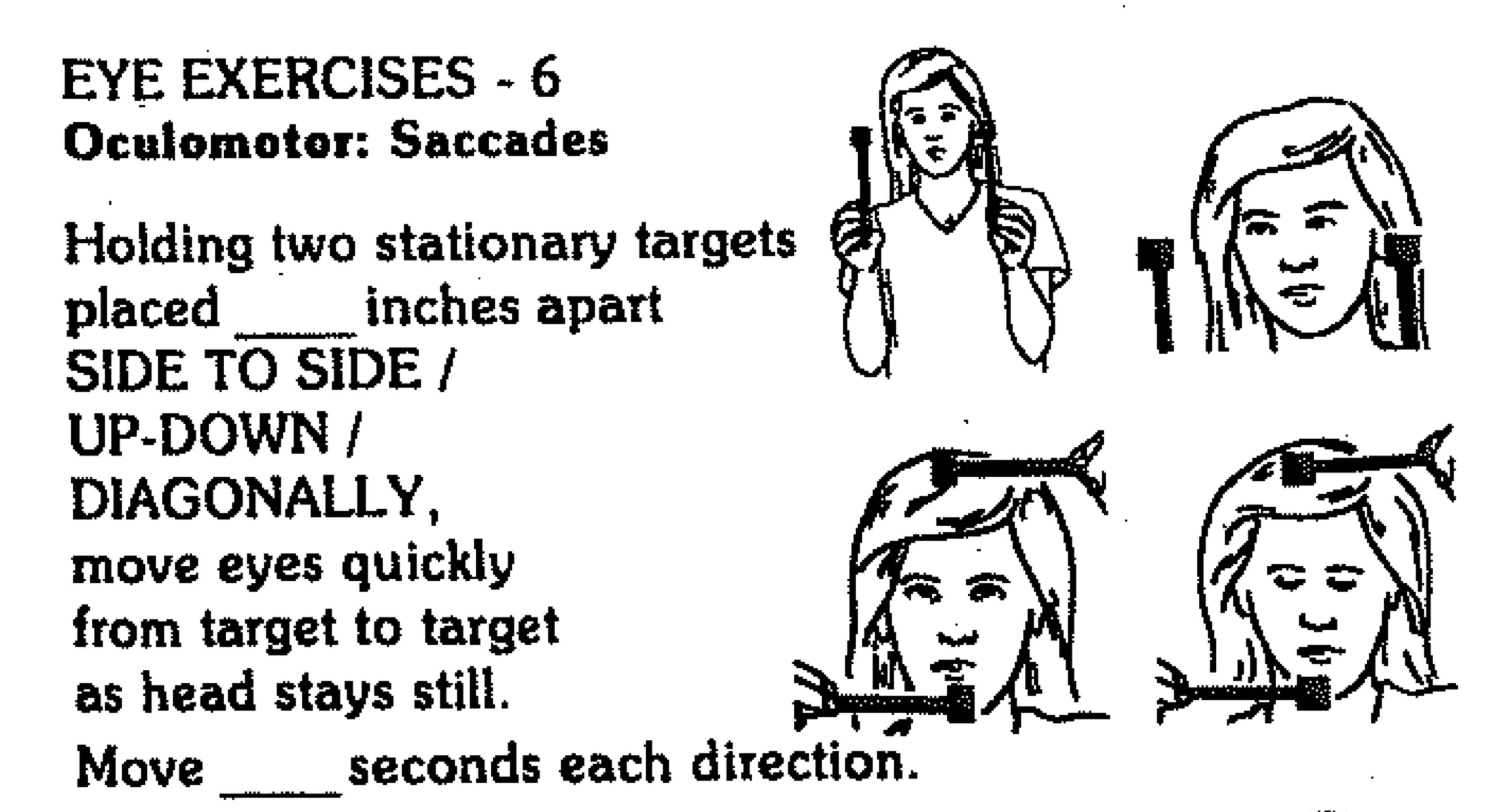


**Exercise #2 (Saccades):**

Keep pens *** apart

- 2 x 45 sec side/side
- 2 x 45 sec up/down


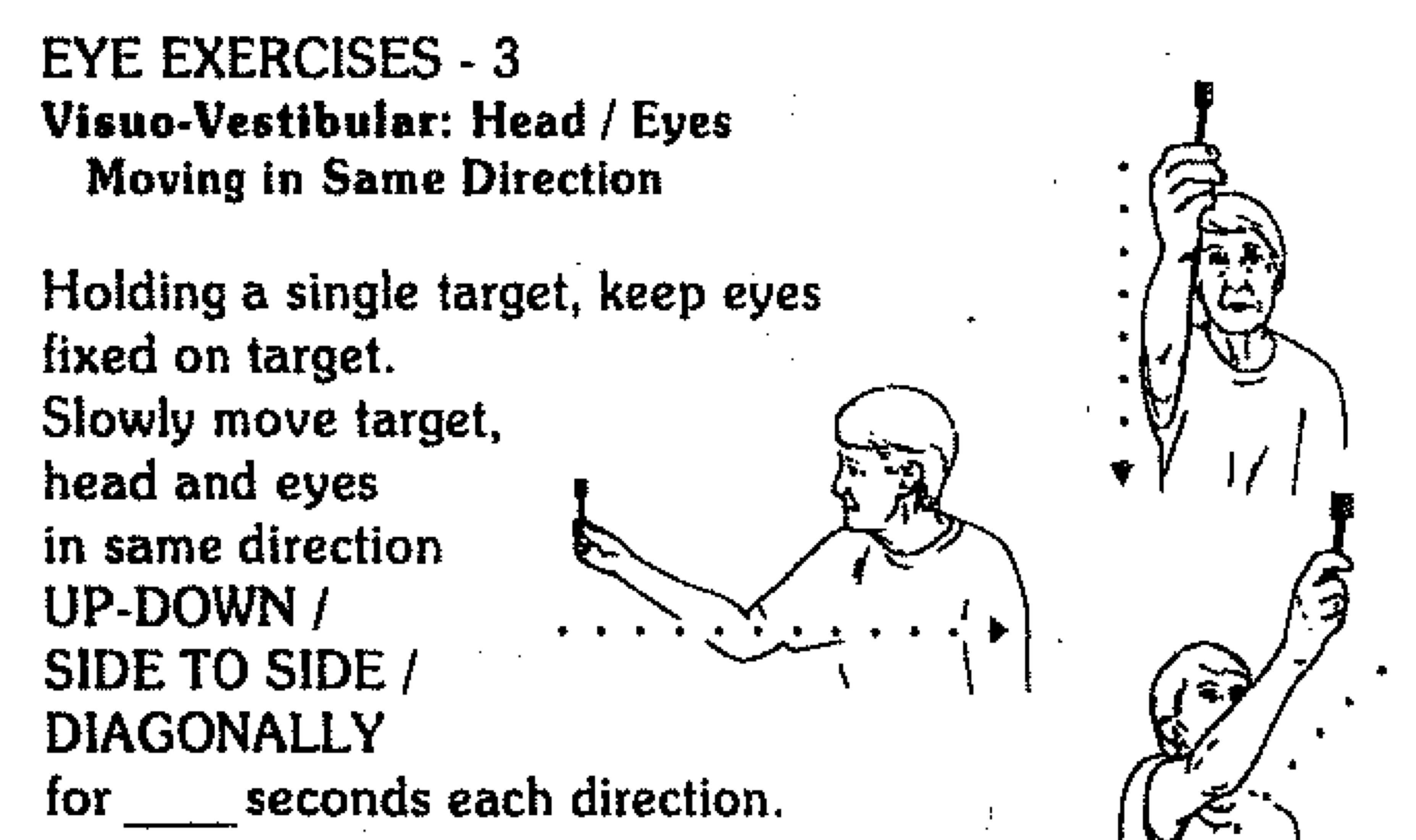


**Exercise #3 (Visual Motion Sensitivity):**

- 2 x 45 sec side/side
- 2 x 45 sec up/down

**Exercise #4:**

**A)** **B)**


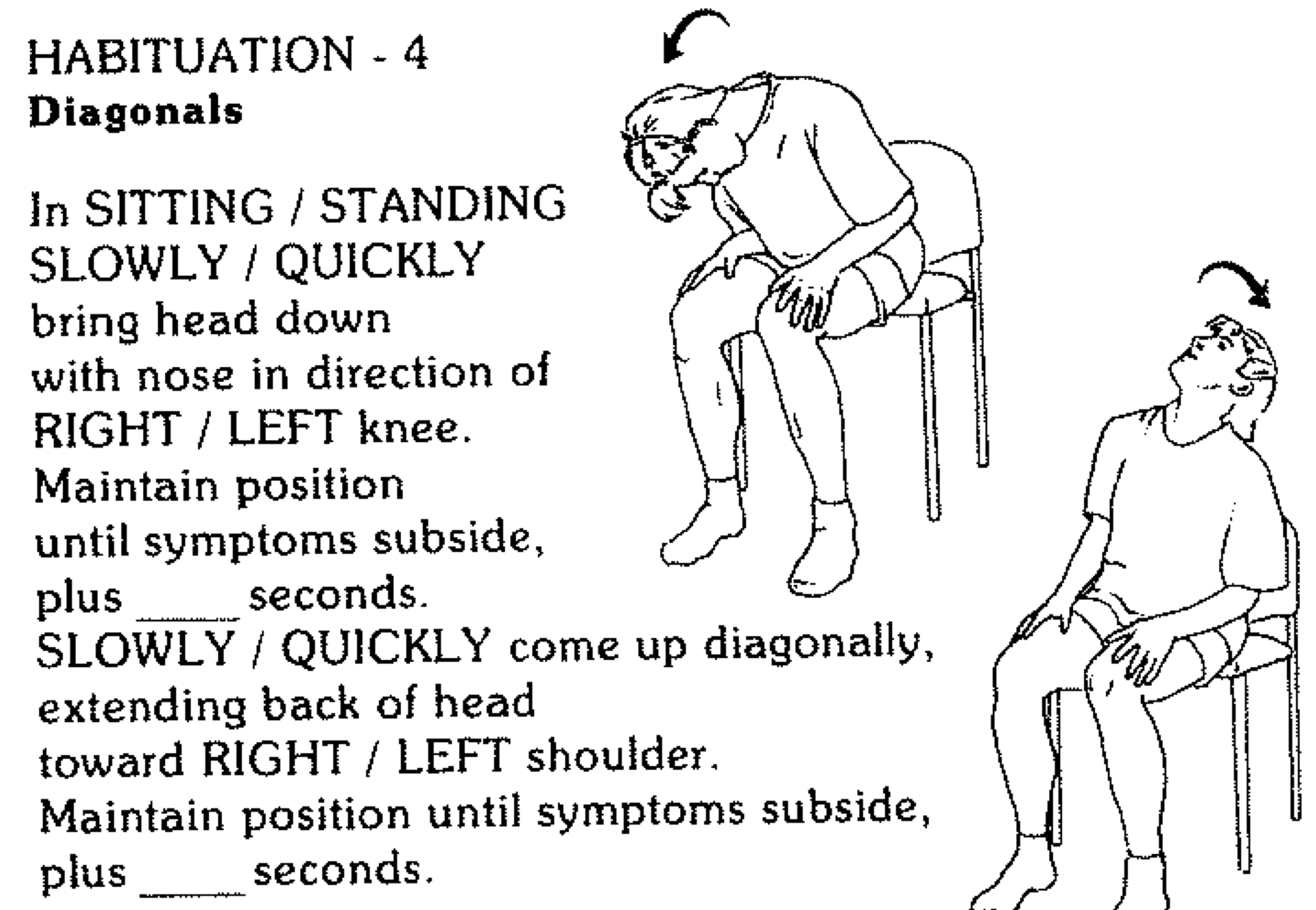

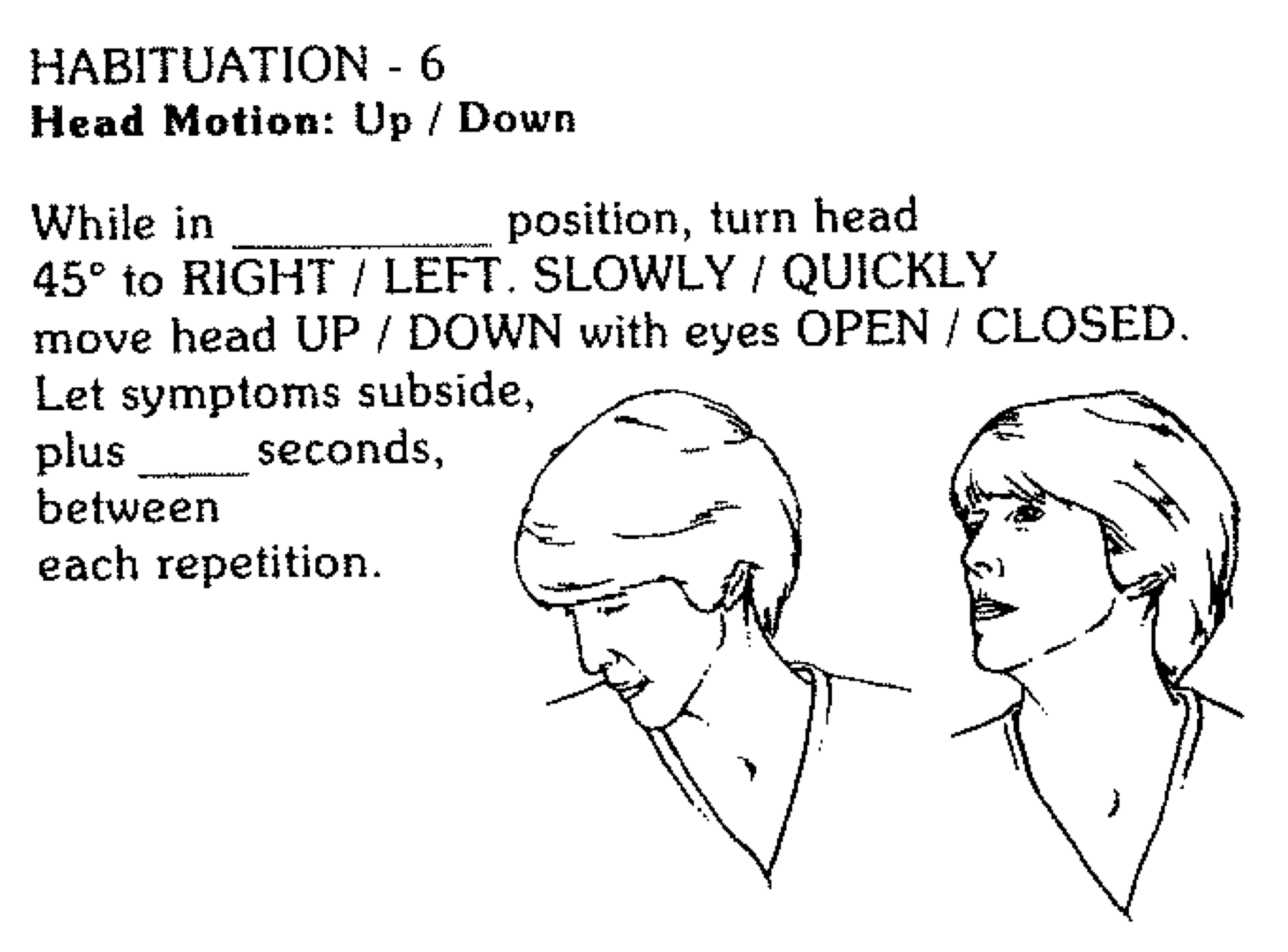


**C)** **D)**


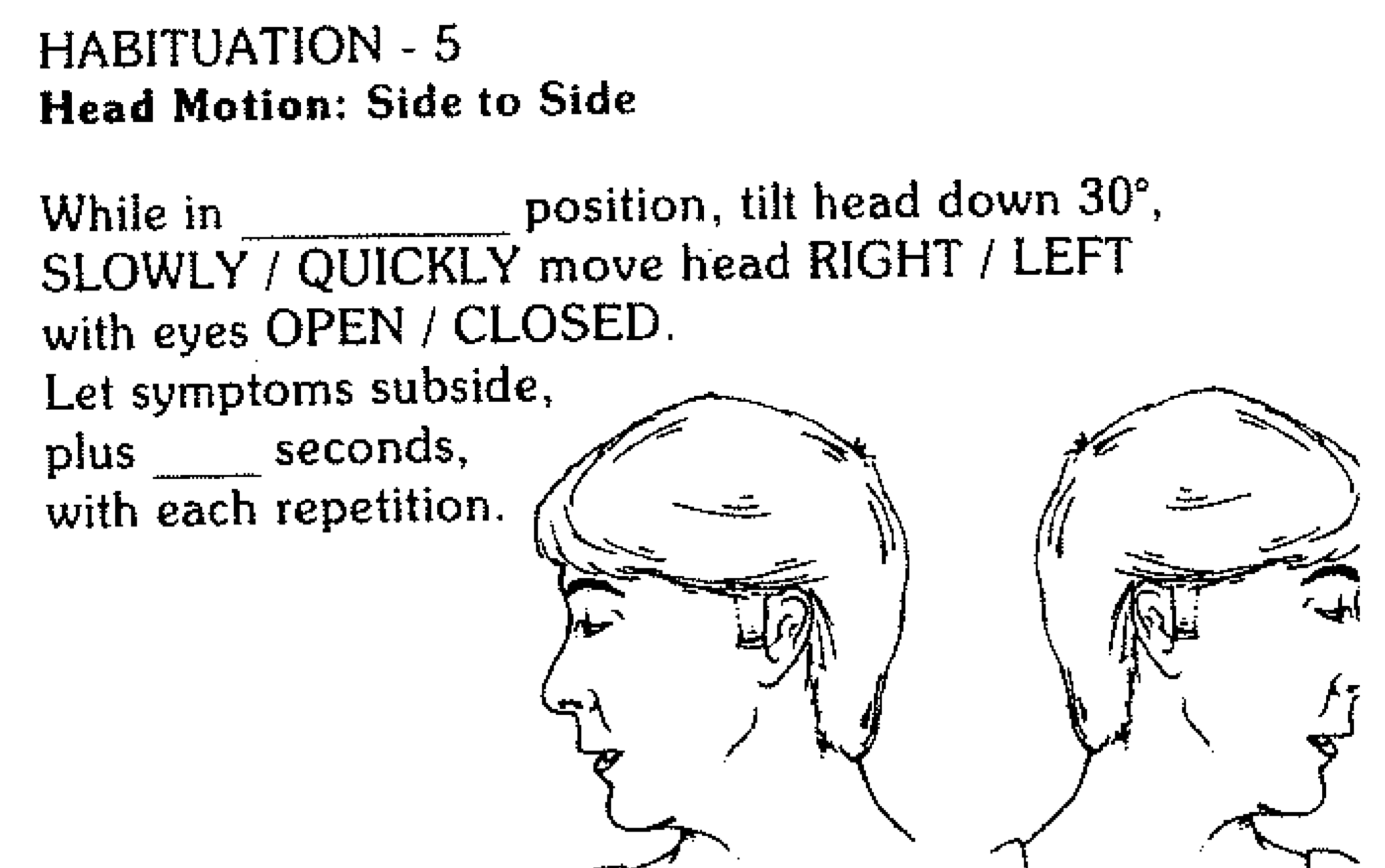

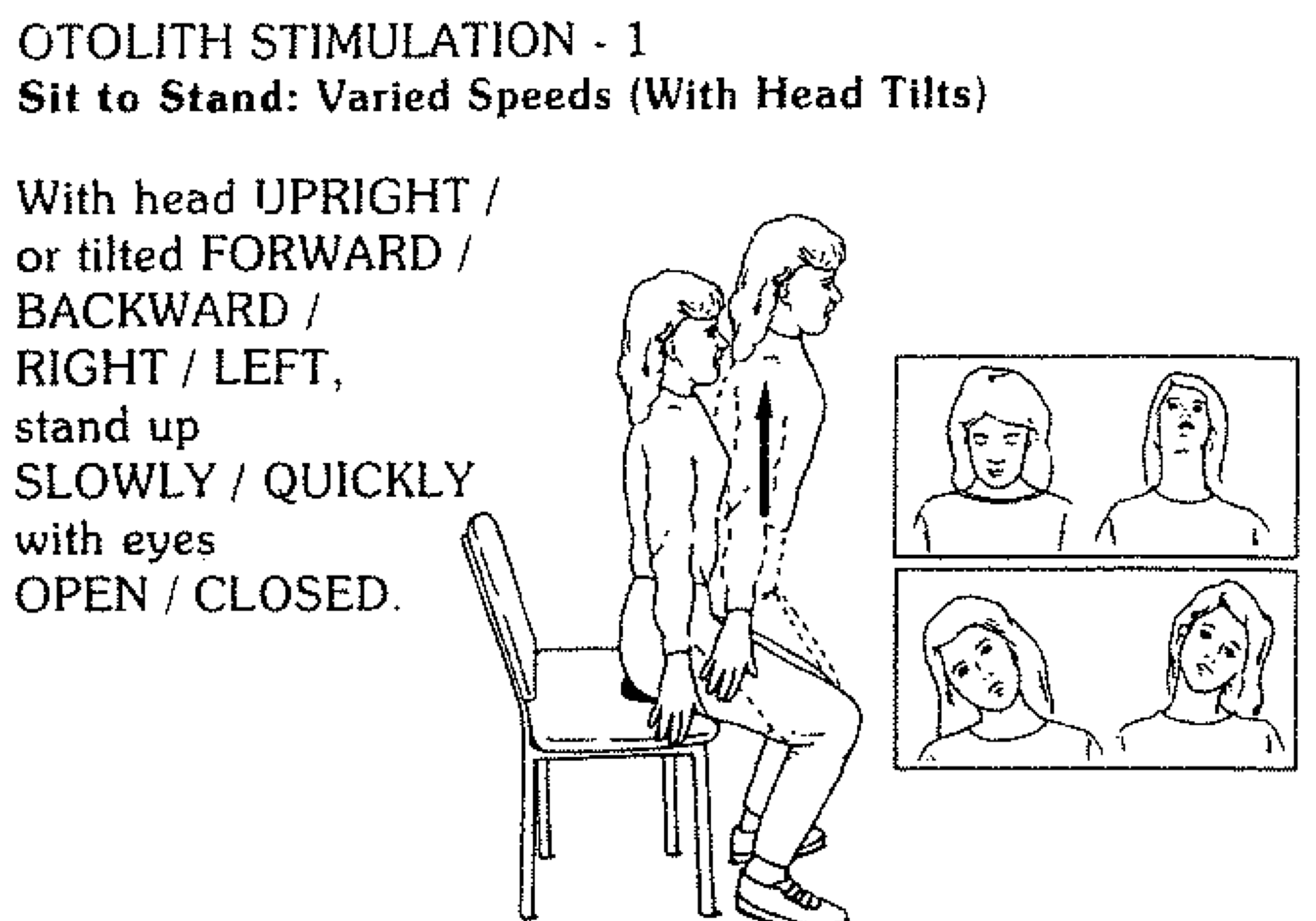


**Exercise #5: Cardio**

- Time: 15-20 minutes
- Intensity: Subthreshold, meaning concussion symptoms may increase 0-2 points on 0 to 10 scale
- Type: Based on preference and exercise intensity tolerance. See below list:

Options:

Walk

Stationary Bike

Other

# Supplementary Tables

| **Supplementary Table S1:** Spearman’s Rank correlation coefficients for changes from pre-rehabilitation to post-rehabilitation on Post Concussion Symptom Scale (PCSS) total symptom severity score and Dizziness Handicap Index (DHI) total score with changes in objective measurements of balance performance (Sensory Organization Test – SOT and Central Sensorimotor Integration Test – CSMI Test outcomes). | | | | |
| --- | --- | --- | --- | --- |
| **Variable** | **PCSS Total Symptom**  **Severity Score** | | **DHI Total Score** | |
|  | **Spearman’s Rho (95% CI)** | **p Value** | **Spearman’s Rho (95% CI)** | **p Value** |
| SOT Composite Score | -0.09 (-0.45, 0.29) | 0.644 | -0.24 (-0.62, 0.19) | 0.222 |
| **CSMI Condition - SS/EC** |  |  |  |  |
| Vestibular Weighting | -0.12 (-0.48, 0.28) | 0.533 | -0.29 (-0.55, 0.07) | 0.139 |
| Time Delay (ms) | 0.01 (-0.36, 0.39) | 0.977 | -0.18 (-0.5, 0.26) | 0.361 |
| Normalized Stiffness | 0.04 (-0.34, 0.44) | 0.827 | 0.13 (-0.32, 0.52) | 0.498 |
| Normalized Damping | 0.11 (-0.32, 0.5) | 0.577 | 0.05 (-0.38, 0.47) | 0.786 |
| RMS CoM Sway | 0 (-0.36, 0.35) | 0.983 | -0.1 (-0.5, 0.32) | 0.603 |
| RMS Remnant CoM Sway | -0.05 (-0.45, 0.36) | 0.816 | 0.25 (-0.15, 0.56) | 0.198 |
| RMS Internal Sensory Noise | -0.08 (-0.48, 0.33) | 0.673 | ***0.52 (0.14, 0.75)*** | ***0.005*** |
| **CSMI Condition – SS+VS/EO** |  |  |  |  |
| Vestibular Weighting | -0.12 (-0.48, 0.32) | 0.546 | -0.1 (-0.46, 0.32) | 0.622 |
| Time Delay (ms) | 0.02 (-0.37, 0.41) | 0.934 | 0.22 (-0.2, 0.56) | 0.271 |
| Normalized Stiffness | -0.06 (-0.41, 0.33) | 0.775 | -0.15 (-0.55, 0.29) | 0.459 |
| Normalized Damping | 0.05 (-0.35, 0.42) | 0.816 | -0.08 (-0.52, 0.35) | 0.700 |
| RMS CoM Sway | 0.16 (-0.25, 0.5) | 0.422 | 0.18 (-0.24, 0.55) | 0.367 |
| RMS Remnant CoM Sway | 0.26 (-0.19, 0.63) | 0.189 | 0.2 (-0.23, 0.58) | 0.297 |
| RMS Internal Sensory Noise | 0.25 (-0.2, 0.6) | 0.199 | 0.25 (-0.16, 0.57) | 0.197 |
| Italicized and bolded values indicate significant Spearman’s rank correlation (*p* < 0.05).  Abbreviations: CI, confidence interval; EC, eyes closed; EO, eyes open; SS, support surface stimulus; VS, visual surround stimulus; RMS, root mean square; CoM, center of mass. | | | | |

| **Supplementary Table S2:** Spearman’s Rank correlation coefficients for pre-rehabilitation measures of Post Concussion Symptom Scale (PCSS) total symptom severity score and Dizziness Handicap Index (DHI) total score with objective measurements of balance performance (Sensory Organization Test – SOT and Central Sensorimotor Integration Test – CSMI Test outcomes). | | | | |
| --- | --- | --- | --- | --- |
| **Variable** | **PCCS Total Symptom Severity Score** | | **DHI Total Score** | |
|  | **Spearman’s Rho (95% CI)** | **p Value** | **Spearman’s Rho (95% CI)** | **p Value** |
| SOT Composite Score | 0.02 (-0.38, 0.42) | 0.901 | 0 (-0.39, 0.41) | 0.986 |
| **CSMI Condition - SS/EC** |  |  |  |  |
| Vestibular Weighting | -0.12 (-0.51, 0.3) | 0.560 | -0.32 (-0.66, 0.06) | 0.093 |
| Time Delay (ms) | 0.06 (-0.31, 0.4) | 0.776 | -0.25 (-0.58, 0.16) | 0.192 |
| Normalized Stiffness | -0.08 (-0.44, 0.3) | 0.698 | ***0.47 (0.11, 0.72)*** | ***0.011*** |
| Normalized Damping | 0.2 (-0.22, 0.54) | 0.297 | ***0.46 (0.02, 0.73)*** | ***0.014*** |
| RMS CoM Sway | 0.05 (-0.34, 0.42) | 0.795 | ***-0.39 (-0.67, 0.05)*** | ***0.041*** |
| RMS Remnant CoM Sway | -0.25 (-0.56, 0.12) | 0.205 | -0.37 (-0.64, -0.06) | 0.050 |
| RMS Internal Sensory Noise | -0.22 (-0.52, 0.19) | 0.259 | 0 (-0.35, 0.35) | 0.986 |
| **CSMI Condition – SS+VS/EO** |  |  |  |  |
| Vestibular Weighting | 0.01 (-0.38, 0.41) | 0.954 | -0.14 (-0.53, 0.28) | 0.467 |
| Time Delay (ms) | 0.01 (-0.33, 0.36) | 0.944 | -0.19 (-0.51, 0.22) | 0.342 |
| Normalized Stiffness | -0.19 (-0.53, 0.24) | 0.331 | 0.14 (-0.23, 0.49) | 0.465 |
| Normalized Damping | -0.07 (-0.43, 0.35) | 0.722 | 0.06 (-0.37, 0.47) | 0.781 |
| RMS CoM Sway | 0.21 (-0.22, 0.54) | 0.286 | -0.21 (-0.52, 0.16) | 0.280 |
| RMS Remnant CoM Sway | -0.23 (-0.58, 0.26) | 0.240 | -0.13 (-0.48, 0.31) | 0.511 |
| RMS Internal Sensory Noise | -0.21 (-0.56, 0.23) | 0.274 | 0.09 (-0.31, 0.46) | 0.651 |
| Italicized and bolded values indicate significant Spearman’s rank correlation (*p* < 0.05).  Abbreviations: CI, confidence interval; EC, eyes closed; EO, eyes open; SS, support surface stimulus; VS, visual surround stimulus; RMS, root mean square; CoM, center of mass. | | | | |
